# Convolution Network with Custom Loss Function for the Denoising of Low SNR Raman Spectra †

**DOI:** 10.3390/s21144623

**Published:** 2021-07-06

**Authors:** Sinead Barton, Salaheddin Alakkari, Kevin O’Dwyer, Tomas Ward, Bryan Hennelly

**Affiliations:** 1 Department of Electronic Engineering, Maynooth University, W23 F2H6 Maynooth, County Kildare, Ireland; sinead.barton@mu.ie (S.B.); Kevin.ODwyer@mu.ie (K.O.); 2 Insight Centre for Data Analytics, School of Computing, Dublin City University, Dublin D 09, Ireland; salah.alakkari@insight-centre.org (S.A.); tomas.ward@dcu.ie (T.W.)

**Keywords:** Raman spectroscopy, deep learning, denoising

## Abstract

Raman spectroscopy is a powerful diagnostic tool in biomedical science, whereby different disease groups can be classified based on subtle differences in the cell or tissue spectra. A key component in the classification of Raman spectra is the application of multi-variate statistical models. However, Raman scattering is a weak process, resulting in a trade-off between acquisition times and signal-to-noise ratios, which has limited its more widespread adoption as a clinical tool. Typically denoising is applied to the Raman spectrum from a biological sample to improve the signal-to-noise ratio before application of statistical modeling. A popular method for performing this is Savitsky–Golay filtering. Such an algorithm is difficult to tailor so that it can strike a balance between denoising and excessive smoothing of spectral peaks, the characteristics of which are critically important for classification purposes. In this paper, we demonstrate how Convolutional Neural Networks may be enhanced with a non-standard loss function in order to improve the overall signal-to-noise ratio of spectra while limiting corruption of the spectral peaks. Simulated Raman spectra and experimental data are used to train and evaluate the performance of the algorithm in terms of the signal to noise ratio and peak fidelity. The proposed method is demonstrated to effectively smooth noise while preserving spectral features in low intensity spectra which is advantageous when compared with Savitzky–Golay filtering. For low intensity spectra the proposed algorithm was shown to improve the signal to noise ratios by up to 100% in terms of both local and overall signal to noise ratios, indicating that this method would be most suitable for low light or high throughput applications.

## 1. Introduction

Raman scattering is an inelastic light matter interaction that is resonant with molecular vibrational states. This process can be leveraged to probe for chemical information about a sample by use of a laser source. A laser source is focused onto the sample and the resulting scattered light can be collected by a Raman spectrograph in order to construct a spectrum of the resulting chemical information. This can be useful for probing disparate samples, for example, cell biology or material science applications. However, a core drawback of Raman spectroscopy is that it is a weak process [1] due to the Raman scatter being isotropic from the source but the collection methodology is constrained to a fraction of the radiating angles. For this reason Raman spectra are susceptible to having their distinguishing features corrupted or even obscured by noise which can, under constrained circumstances, limit their sensitivity and reduce their efficacy for classification applications. Therefore, integrating rigorous noise removal methodologies into the post-processing software protocols for the aforementioned experimental procedures is beneficial [2].

Noise can be introduced from thermal effects and the digitization process within the sensor, it can also occur as a result of the statistical inconsistencies associated with monitoring an irradiance source [3]. The effect of these noise sources can be alleviated if the system configuration/application has no restrictions in terms of cost or time. However, in many cases this is not practical [4,5,6,7,8]. It must also be noted that even with an optimal Raman spectrometer and the most carefully designed experimental procedure, a noisy Raman spectrum is unavoidable for many sample types. Therefore the most flexible method of reducing the effect of these noise sources is through software post-processing.

Savitzky–Golay (SG) filtering [9] is a post-processing technique that is commonly used to filter spectra in order to reduce the impact of noise on statistical classification [10,11]. This filtering technique works by dynamically fitting a polynomial to consecutive windows of data points in the spectrum in order to approximate the shape of the spectrum in the presence of a randomly varying noise signal. Under certain conditions, this can have a negative impact on spectral features, especially sharp local features or ’peaks’, in particular, high noise/low light applications that require high levels of smoothing. Identifying the optimal polynomial order and window that optimally smooths a given dataset with a given Signal-to-Noise Ratio (SNR) can also be cumbersome; e.g., a spectrum with a high SNR should be smoothed using an SG filter with a window size only sightly greater than the polynomial order in order to preserve the peak formations, while a spectrum with a low SNR would require a large window to effectively smooth the noise. For this reason, a trade-off is required between peak preservation (higher polynomial and relatively small window size) and effective noise smoothing (lower polynomial and relatively large window size). Therefore, the implementation of a denoising technique that simultaneously smooths while maintaining peak fidelity, without the need for initial testing/optimisation, is highly desirable.

In recent years, deep learning Convolutional Neural Networks (CNNs) have been shown to perform well at denoising coherent Raman spectra when compared to established denoising techniques [12,13,14]. In these contributions deep learning was applied to three-dimensional hyperspectral image sets. Recently, a 1-D CNN was developed to denoise Raman spectra using a standard loss function [15] and was shown to outperform wavelet denoising for a range of samples. The primary difference between the model proposed in this paper and previously developed neural network solutions is that, in this instance, a custom loss function is used in order to balance the networks prioritization between overall improvement in spectral SNR and the preservation of important spectral peaks that facilitate the identification of varying samples. This is a key contribution which has a significant impact on performance. Furthemore, in order to improve the general applicability of the network so that it may be used with spectra obtained from diverse sources, the algorithm was trained on large datasets of simulated Raman spectra with randomised spectral profiles. Testing was performed on experimental data. These simulated datasets included spectra containing only sharp features, which are similar to spectra recorded from pure chemicals, as well as spectra containing peaks of highly varying width and amplitude, which are similar to spectra recorded from biological samples. The results presented in this paper, clearly demonstrate that by training the CNN with a custom loss function sensitive to Raman features significant for chemical characterization, there is a clear improvement in the performance of the network with respect to denoising than compared with using the traditional L2 Norm.

The paper is split into four main sections. Section 2 provides an overview of the properties of the chosen neural network and custom loss function. Section 3 defines the custom loss function. Section 4 deals with the simulated spectral datasets that are used to train the network and also specifies the training parameters of the network. Section 5 provides details on the experimental data and in Section 6 the results of the proposed algorithm are provided and are compared with two SG smoothing algorithms, one designed to prioritize smoothing and the other designed to prioritize peak preservation.

## 2. Theory

Deep learning has become a widely used practical approach to solve a plethora of research problems in different areas including computer vision, natural language processing and signal processing. Many deep learning architectures have been deployed for image recognition tasks based on the use of convolutional layers as the main building block. Two reasons for the success of CNNs are their high efficiency in capturing low-level details in the input samples and their highly-parallel nature which facilitates their execution on Graphical Programming Units (GPU) more so than conventional fully connected networks. For this reason, CNNs are chosen as the algorithmic basis for the proposed denoising algorithm.

### 2.1. Convolutional Neural Networks

Unlike fully-connected neural networks where each unit in a layer depends on the entirety of the units in the previous layer, a CNN regularizes spatial patterns using a moving window type of processing. The convolutional layers of CNNs are spatially invariant, which can be useful for similar features that appear in different areas such as Raman peaks. In this case, each unit in a layer depends on units in the previous layer within a specific patch. The moving window consists of multiple filters (also referred to as kernels or convolutions) where each filter contains weights for the corresponding patch. The window moves through the entire image with a specific stride. After computing the convolutions, the resulting feature map is normalized using batch normalization [16]. After that, the activation function is performed on the normalized feature map. The activation function is usually a rectified linear unit (ReLU) defined as follows f(x)=max(0,x). Such activation was found to be advantageous compared to the sigmoid function in terms of minimizing the loss function [17]. Pooling layers may also be used in CNNs. These layers operate a moving window in which a single value is chosen per patch per kernel. Two types of pooling layers are commonly used, max pooling and average pooling. The output of each convolutional layer is referred to as a feature map. The final layers usually follow the structure of fully-connected MLP. Similar to the conventional MLP, CNNs are also trained using the back-propagation learning algorithm in which convolutional gradients are derived using the chain rule.

#### 1D-CNNs for Denoising Tasks

In addition to computer vision and image processing tasks, deep convolutional networks have also been applied to many signal processing applications including signal denoising tasks. Only a number of representative and relevant papers are discussed here. Liu et al. studied deep learning networks for speech denoising and compared their results with non-negative matrix factorization (NMF) [18]. Their results showed that the deep learning model provided significantly better performance. However, only fully connected deep learning models were investigated. Park and Lee investigated the performance of CNNS for hearing aid systems and compared them against recurrent neural networks [19]. They proposed a convolutional deep learning architecture based on multiple convolutional units referred to as Recurrent-Convolutional Encoder Decoder network (R-CEC). Not only was the performance better than the recurrent network model but it also required significantly less memory, making the model suitable for deployment as part of an embedded system for hearing aids.

Raman spectral denoising falls into this class of 1-D processing. In general, the problem of signal and speech denoising is to generate a clean version of the signal from a noisy one. This can be achieved by simply training a deep convolutional network on signal pairs. One of the pair is a clean synthetic signal and the other is the same synthetic signal but with appropriately modeled noise added. This is known as a supervised learning problem. In this case, the deep learning model will try to find a suitable mapping that transforms a noisy signal to its original clean version. The gradient descent optimization (or its variants such as ADAM optimization) are used to find such a mapping. This requires defining a loss (error) function based on the training samples and the application of a gradient descent algorithm to find the optimal solution that minimizes the loss over all the training data. For signal denoising problems, the $L_{2}$ Norm (or Mean-Squared-Error) between the resulting transformed signal and the original one is commonly used as the loss function. In this paper, however, we introduce a loss function that is more sensitive to the information-bearing structure of Raman spectral data. When used in conjunction with an appropriate neural network, it results in excellent performance compared to existing methods. Most applications of CNNs involve image processing and are inherently two-dimensional in design. Since in the case of Raman denoising we are dealing with one-dimensional (1D) signals, the convolutional network will be relatively simple.

## 3. Establishing a Non-Standard Loss Function

SNR is an important metric for establishing signal quality in all fields of engineering and a modified definition of SNR is employed here to design a custom loss function, as well as to evaluate the performance of the proposed algorithm.

### 3.1. Signal to Noise Ratio and the SNR Product

SNR may be defined as the ratio of the maximum value of the intensity of the spectrum across the wavenumber range of interest to the Root Mean Square Error (RMSE) of the noise present in the spectrum [20]. It is possible to estimate the noise level through a comparison between the spectrum in question, $x^{e}$, and a low noise reference spectrum, $x^{ref}$. This definition of SNR and the procedure used to calculate it are defined as follows:

(1a) $$\begin{array}{cc} SNR(x^{e}) & =\frac{\max(x^{ref})}{RMSE(x^{e},x^{ref})} \end{array}$$

(1b) $$\begin{array}{cc} RMSE(x^{e},x^{ref}) & =\sqrt{\frac{1}{N}\sum_{i=1}^{N}{\left(x_{i}^{e}-x_{i}^{ref}\right)}^{2}} \end{array}$$

where max() is a function that returns the maximum value in the input vector and both spectra are of length *N*. Going forward, this is referred to as global SNR for the remainder of the manuscript. However, while smoothing may increase the global SNR of a spectrum it can also negatively affect sharp local features, which are generally of more importance for subsequent multivariate statistical classification of the spectra. In order to evaluate the performance of the denoising algorithm specifically on sharp spectral features, the SNR is also calculated specifically in the neighbourhood of a peak region. More specifically, the SNR is calculated in a 2n+1 sample window of $x^{e}$ that is centered on the most prominent peak, which has been identified to be centered at index pk. The 2n+1 sample window is determined based on the Full Width Half Maximum (FWHM) of the peak in question. The SNR for this peak region is defined as follows.

(2) $$SNR\left(x^{e}\left[pk-n:pk+n\right]\right)=\frac{\max(x^{ref})}{RMSE(x^{e}\left[pk-n:p+n\right],x^{ref}\left[pk-n:pk+n\right])}$$

Let it be noted that, in order to provide a consistent comparison between global and peak SNR, the same maximum value is used in both Equations (1) and (2). Both distinct SNR measurements may be combined into a single metric so that the evaluation of the overall performance of the algorithm can be contained in a single calculation known as the SNR product [21] as described in the following:

(3) $$SNR_{prod}=\frac{SNR(x^{e})}{SNR(x)}\times \frac{SNR(x^{e}\left[pk-n:pk+n\right])}{SNR(x[pk-n:pk+n])}$$

where *x* is the raw noisy spectrum before denoising and $x^{e}$ represents the denoised spectrum. The global and peak SNR values of *x* may also be obtained using Equations (1) and (2). In the case where the algorithm negatively affects peak fidelity or alters the underlying structure of the spectrum, the SNR product may return a value <1. In the case that the denoising method improves the SNR of the spectrum, the value returned from the SNR product will be >1.

### 3.2. The Custom Loss Function

The loss function plays a critical role in training deep learning models. It provides a metric to evaluate model performance and the choice of the metric can be application dependent. In signal denoising tasks, the ${Ł}_{2}$ Norm or Mean Square Error is commonly employed as the loss function. This can be defined as follows:

(4) $$MSE\left(x^{e},x^{ref}\right)=\frac{1}{N}\sqrt{\sum_{i=1}^{N}{\left(x_{i}^{e}-x_{i}^{ref}\right)}^{2}}$$

where $x_{i}^{ref}$ is an observed sample from the target signal, $x_{i}^{e}$ is the predicted value for this sample using the deep learning model and *N* is the total number of samples in the vector. During the training phase, the deep learning model adjusts its inner weights so that the MSE is reduced towards zero. While this loss function has been extensively used in regression and denoising tasks, recent studies suggest adding the structured similarity index (SSIM) to enhance the quality of the recovered signal [13,14]. One point to consider in the Raman denoising problem is that the dominant peaks in the signal are of high importance in analyzing the material structure. Therefore, it is essential to ensure that such peaks are appropriately preserved in the denoised signal. One problem that may occur when using the $L_{2}$ Norm is the fact that higher priority might be given to denoising areas of limited spectral information (regions of low intensity variation). While removing such a type of noise is essential, one needs to consider the noise in the area of a spectral peak (i.e., a region of high intensity variation). To the best of our knowledge, this problem has not been addressed in the literature. The SNR product, which we have introduced, provides a suitable solution to this problem. However, such a non-linear function is difficult to optimize due to the fact that it is unbounded and results in stability problems when using stochastic gradient descent optimization or its variants. Hence, we propose a more practical solution by simply adding another term to the MSE loss. This term corresponds to the MSE computed at a local region occupied by the most prominent peak. The new loss function is described as follows:

(5) $$Loss\left(x^{e},x^{ref}\right)=MSE\left(x^{e},x_{ref}\right)+\alpha MSE\left(x_{peak}^{e},x_{peak}^{ref}\right)$$

where $x_{peak}^{e}$ and $x_{peak}^{ref}$ are the samples located in the region around the single most prominent peak (identified easily by the maximum value of the spectrum) in the spectrum and α is a weighting factor that determines the relative priority that the algorithm places on smoothing versus peak preservation during the training process, which is discussed more in the following sections.

## 4. Network Architecture and Training Settings

### 4.1. Simulation of Raman Spectra for Training

The Neural Network was trained using a dataset of simulated noise-free Raman spectra $X^{ref}$. The susceptibility response of a single Raman transition can be described by a Lorentzian lineshape described as follows:

(6) $$\begin{array}{c} \chi \left(\omega \right)=\frac{A}{\Omega -\omega -i\Gamma } \end{array}$$

where *A* is peak amplitude, Ω is resonant frequency, Γ is the Raman linewidth and ω is the wave number. The spectra are constructed as a sum of a random number of such functions with random frequencies, amplitudes and linewidths. By modifying and randomising the peak amplitude, width and location in the spectra, it is possible to generate a training dataset of simulated spectra that can vary from isolated distinct peaks, which are analogous to chemical spectra, to a complex mix of overlapping peaks that are more analogous to biological spectra. As simulated spectra are not constrained to a specific type of spectral profile, it was possible to train the algorithm as a general purpose denoiser that would be capable of denoising multiple spectral profiles.

**Figure 1:**
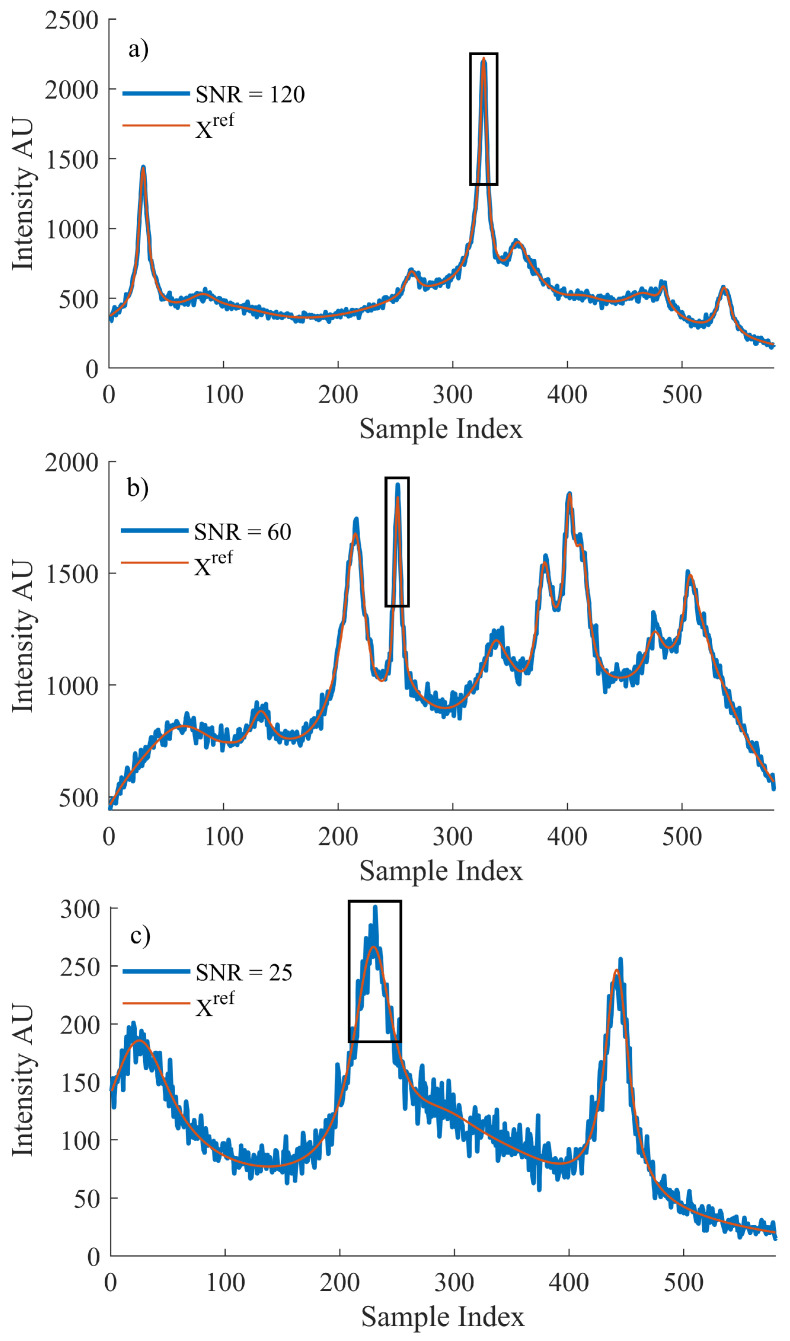
An example of the simulated data used to train the proposed algorithm. The original spectrum, $X^{ref}$, that was produced using Equation (6) and the spectrum after simulated Poisson noise were introduced where the SNR for each resulting spectrum is (**a**) 120, (**b**) 60 and (**c**) 25, respectively. The peak formations that were selected for training the algorithm for the local MSE are outlined by a black box in reach case.

The reference Raman dataset, $X^{ref}$, was then used to generate a matching noisy dataset, *X*, with both datasets then used for training. Raman spectral noise has its own distinct characteristics, which can be modeled and artificially added to the reference dataset. Shot noise is described as the discrepancy between the incident irradiance and the collected intensity. It can be modeled by a Poisson distribution for which the mean value is dependent on the collected intensity at a particular wavenumber index, *i*. Each individual wave number index is input to the ’poissrnd‘ function in MATLAB and the resulting value is used to simulate the effect of shot noise in the system [5]. For this reason, the noise added will vary based on intensity of the simulated spectrum, which is programmed to vary randomly between 0 and 4000. In this manner, it is possible to create large datasets of randomised spectral profiles and SNRs that may be used to train the network. The maximum range of SNRs produced using this method is the following: 15<SNR<145. Figure 1 depicts three example spectra from different SNR levels to highlight the broad range of spectra that the method is capable of producing.

Let it be noted that the peak region highlighted in Figure 1 is defined by the FWHM of the most prominent peak within the spectrum, as detailed in the discussion contained in Section 3.1 related to the use of Equation (2).

### 4.2. Network Architecture

The proposed architecture is described as a fully convolutional deep neural network for denoising. The proposed model accepts a low SNR Raman spectrum of 600 samples as input and the goal is to generate a denoised version of the signal.

The model consists of five convolutional layers, each of which have a stride of one and operate in same-padding mode so that the resulting signals in the feature map will have the same width (number of samples) as the input signal. It is acknowledged that fewer convolutional layers are used in comparison to similar network structures [19]. A larger number of layers was tested but did not enhance the performance. Each convolutional layer is followed by batch normalization and ReLU activation layers, with the exception of the final convolutional layer. Details of the different convolutional units of the network are provided in Table 1. Pooling layers or fully-connected MLP were not considered for the proposed model.

**Figure 2:**
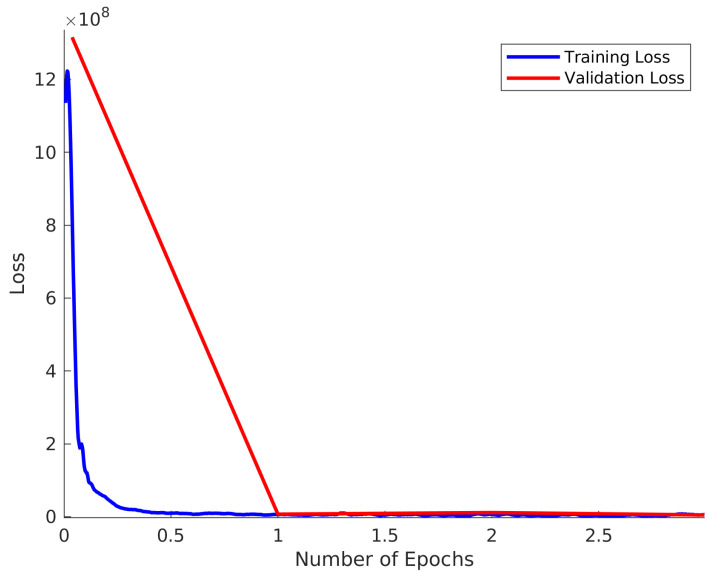
Training and Validation loss for α=50.

Hyperparameter tuning for the learning rate was performed using the grid search technique. An initial learning rate of $10^{-9}$ was input to a stochastic gradient descent optimization with a momentum of 0.9 in order to train the model. The value of 0.9 was chosen as it is a commonly accepted default value for this optimization method in the literature. During the training phase, the model received training examples in mini-batches of size 128 examples/minibatch. Size 128 in this case represented a good compromise between speed of convergence and performance, although we recommend to the adopters of our methodology to experiment with this parameter for their own datasets. The gradient descent update is performed after each mini-batch and each one that passes through the entire dataset (comprising multiple mini-batches) is referred to as a training epoch. The presented model is trained for 100 epochs where the learning rate is halved after 50 epochs in order to enhance the convergence as dictated by convention. The majority of the learning takes place in the first few epochs, which is illustrated in Figure 2.

**Figure 3:**
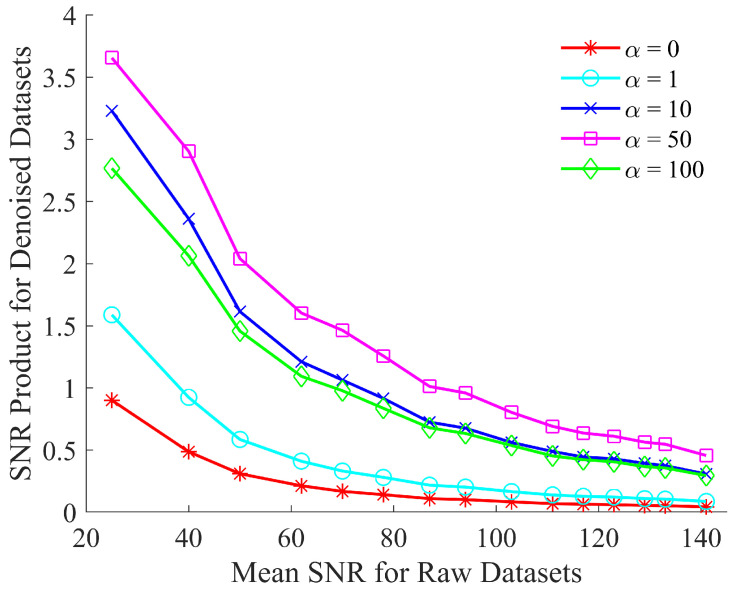
Optimizing the SNR product of CNN denoised spectra by evaluating the custom loss function, defined in Equation (5), for α=0,1,10,50,100.

A further optimization step was required to establish the optimal weighting of the local vs. global MSE. In order to perform this, the value of α was changed to five distinct values, i.e., 0, 1, 10, 50 and 100 and, in each case, the proposed algorithm was used to denoise the same test dataset of simulated spectra. The resulting denoised spectra were then examined in terms of the SNR product to establish the optimal weighting. The results of this analysis are depicted in Figure 3.

Figure 3 shows that the performance of the network improves through the use of the custom loss function. This improvement is due to the enhanced peak fidelity of the denoised spectrum when compared to the reference spectrum. The improvement in the resulting SNR product ceases to increase beyond α=50. Therefore, the custom loss function is fixed at this value of α for the results shown in Section 6.

The training loss and validation loss is shown in Figure 2 for the case of α=50. In this case, 10,000 spectra were used in both the training sets and validation set. The validation loss is taken to be the average loss of the validation set calculated at the end of each epoch. The training loss is plotted for each of the 79 batches in each epoch and this is smoothed using a Savitsky–Golay filter of window size 29 and polynomial order 3. This result clearly demonstrates that there is no over-training of the model. It is also notable that the majority of improvement in network performance happens within the first epoch, although slow improvement is observed over the entire range.

## 5. Data Collection

Effective testing of denoising algorithms requires spectra that have been collected under rigorous and highly controlled experimental procedures so that the SNR can be reliably calculated using the definitions given. For this reason, biological samples are unsuitable as they are non-homogeneous and prone to photo-bleaching over long exposures. Based on the randomized spectral profiles used in training and detailed in Section 4.1, it is expected that the algorithm is suitable for use on any spectral dataset including biological spectra. However, in order to test the algorithm on experimental data in a manner that satisfies the definition of SNR provided in Equation (1), an alternative sample that produces a spectral profile similar to that of a biological spectrum without the aforementioned drawbacks of investigating a biological sample is required. Therefore, spectra were recorded for testing from a PMMA plastic sample in the form of a micro-fluidic slide from Ibidi GmBH. Polymers, such as the micro-fluidic slide, are not affected by the aforementioned drawbacks although the spectra obtained from them exhibit spectral features that may be considered analogous to those obtained from of biological samples. More details on this sample can be found in Ref. [22]. By recording experimental data from this source, it is possible to ensure that the performance of the CNN algorithm is assessed on a sample that produces a spectral profile with similar features to a cell spectrum while also reducing the experimental variability. The thermal stability of the polymer in question also facilitates the acquisition of a high SNR spectrum that can be used as benchmark with which to estimate the experimental noise in short-acquisition spectra.

The experimental system that was used to acquire the spectra has been detailed in previous publications by the research group and so the specifics of the Raman spectrometer may be found in Ref. [4]. A low Numerical Aperture (NA) microscope objective (Olympus UMplanFl 4x/0.1) was used primarily due to its large depth of field, thereby minimizing the impact of system drift over the course of an extended experiment. The polymer also inherently produces a strong Raman response and therefore the low NA also enabled the system to record spectra in the low SNR range for the purposes of this paper. A low SNR level is considered to be where the noise has the potential to obscure or prevent reliable reconstruction of small peak formations. A low SNR value would be considered to be <50. Six datasets, comprised of fifty spectra each were recorded using this method. Distinct mean SNR values for each dataset were achieved by specifying an acquisition time for each dataset and maintaining the other system parameters. A low noise reference spectrum to be used as $x^{ref}$ was recorded using an extended exposure and the spectra were standardized for comparison to this reference through the use of an Extended Multiplicative Signal Correction (EMSC) algorithm [23,24].

## 6. Experimental Results

**Figure 4:**
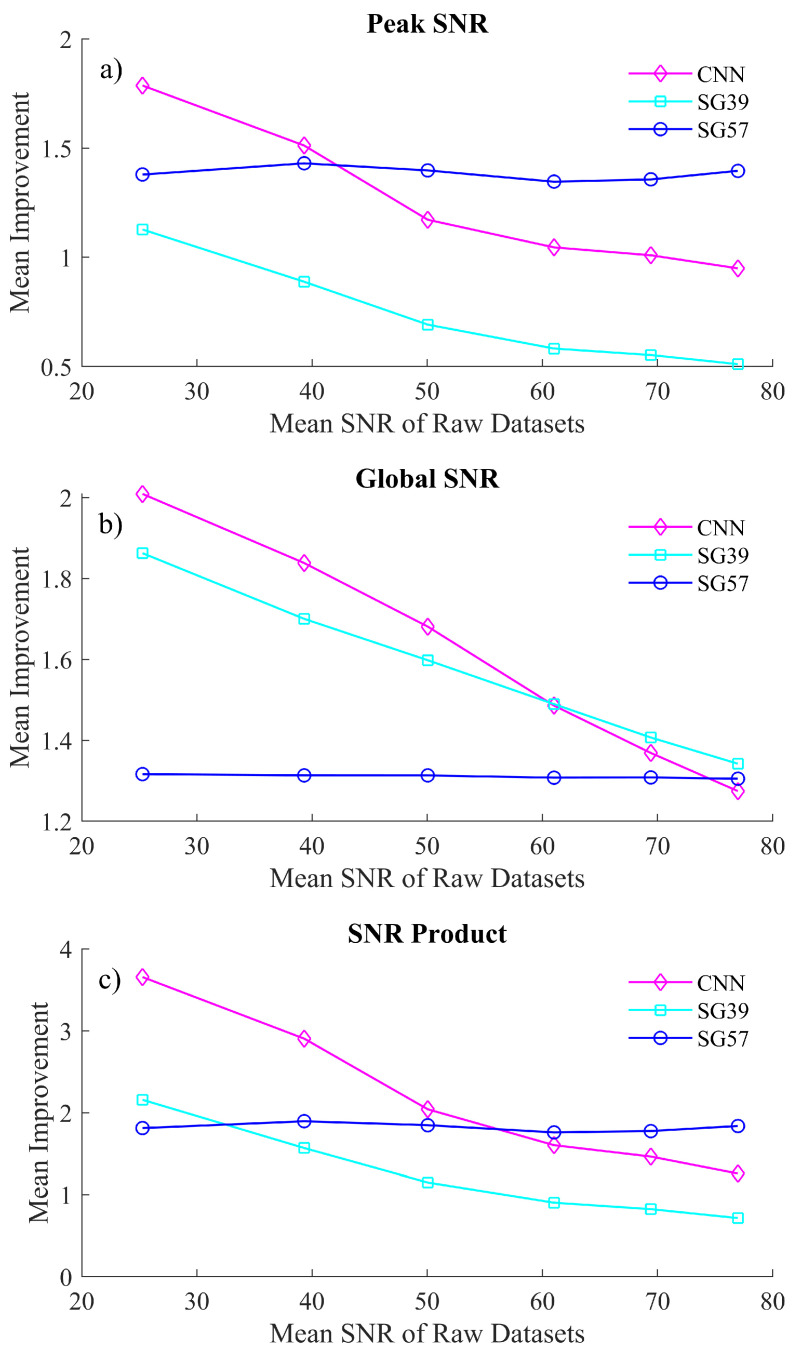
A comparison of the mean improvement in (**a**) global SNR, (**b**) peak SNR and (**c**) the SNR product from the the proposed CNN denoising algorithm and the competing SG filters. The *x*-axis is labeled in terms of the mean SNR of the raw datasets collected under the experimental conditions detailed in Section 5.

The datasets that are detailed in the previous section were denoised both by the CNN denoiser and by two distinct SG filters chosen from an array of SG filter parameters that were evaluated to be most competitive to the proposed algorithm in terms of peak fidelity and overall SNR, particularly in the low SNR ranges. An SG filter of polynomial 3 and window of 9 (SG39) was selected, which produces denoised spectra with comparable global SNR with respect to the proposed algorithm; however, this SG filter smooths the peak regions more than the proposed algorithm. Similarly, an SG filter of polynomial 5 and window 7 (SG57) was selected, which preserves sharp features in the spectrum [21] and, therefore, produces denoised spectra with comparable peak SNR with respect to the proposed algorithm; however, this SG filter is naturally less effective at denoising smooth regions than the SGH39 filter as well as the proposed algorithm. Both sets of resulting denoised spectra were evaluated in terms of global SNR (Equation (1)), peak SNR (Equation (2)) and the SNR product (Equation (3)). A single sharp local feature was chosen to benchmark peak fidelity in the spectra. This feature is centered at $896.2\;{\mathrm{cm}}^{-1}$, chosen since its formation is visually similar to the phenylalanine peak in biological spectra and was investigated across a window of 13 samples, i.e., the maximum point and the surrounding ±6 samples. The results of this analysis can be observed in Figure 4. Figure 4a,b show the relative SNR improvement for various noisy datasets for peak regions and for the entire (global) spectrum, respectively.

The CNN algorithm shows a significant improvement of the SNR product, in particularl, for very noisy spectra, i.e., spectra that have an SNR of below 50. While SG57 exceeds the CNN performance for spectra with SNR > 40 in terms of peak fidelity, the CNN significantly outperforms SG57 in terms of global SNR for almost all datasets. For the second SG filter, the CNN algorithm outperforms SG39 in almost all cases of both peak and global SNR. In terms peak SNR, the CNN is significantly superior to SG39 (with the latter showing an SNR improvement <1 for the majority of the datasets, indicating that it is negatively affecting the peak formations), while in the case of global SNR there exists only a marginal difference in the two methods.

**Figure 5:**
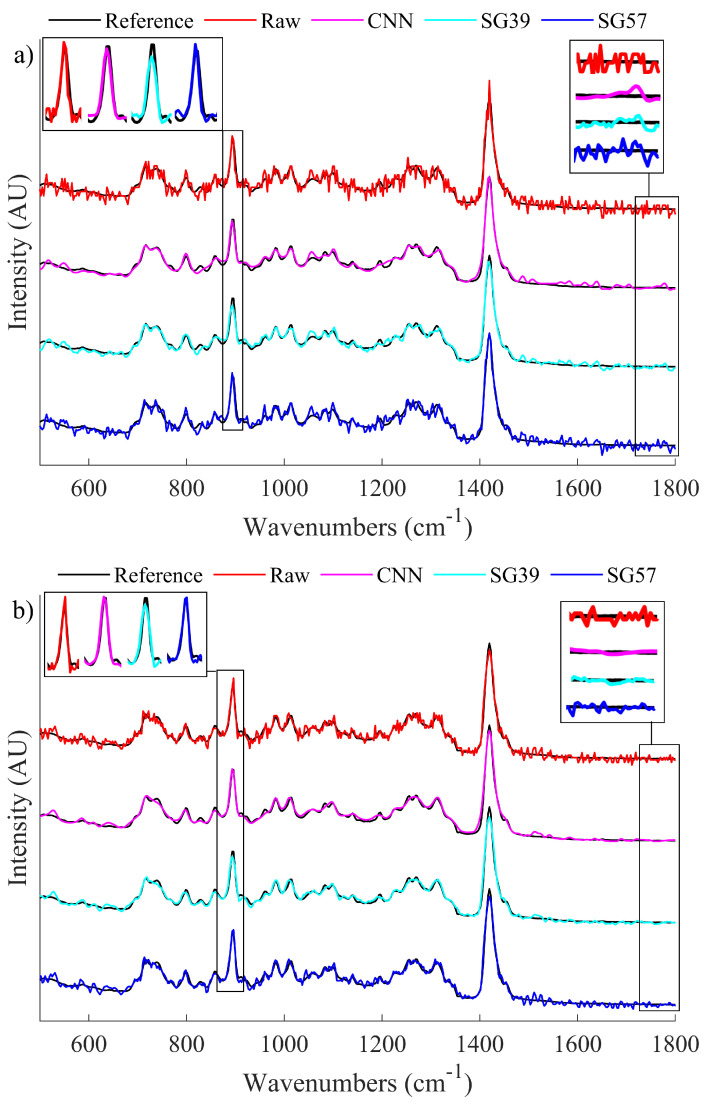
A visual comparison of the denoising properties of the SG filters when compared to the CNN and the raw data. Areas of interest, i.e. a sharp local peak and an area of low frequency noise, have been enlarged to highlight the difference in performance. (**a**) Illustrates a sample spectrum of raw SNR 25 as well as its denoised counterparts and (**b**) Illustrates a sample spectrum of raw SNR 40 as well as its denoised counterparts.

Clearly the SG filters can each operate comparably to the CNN in one context while performing less effectively in the second context; i.e., the two SG filters are not effective at balancing denoising of both sharp and smooth spectral features. This is illustrated using a single metric, the SNR Product metric, which is shown in Figure 4c. This metric demonstrates that the CNN is able to balance both peak and global denoising most effectively for the case of low light spectra. A visual representation of how these processing methods affect the spectra is shown in Figure 5. In Figure 5a, we show the results of denoising a low SNR polymer spectrum (SNR = 25) and in Figure 5b we show results for denoising a relatively higher SNR spectrum (SNR = 40). In both cases, the raw spectrum is shown in red and the low noise irradiance is shown in black recorded over a long acquisition time. The results of the CNN denoiser is shown in pink and the two SG filters are shown in cyan and blue. Two regions are highlighted corresponding to a sharp spectral peak as well as a smooth region of the spectrum.

From the enlarged sections of the spectra around the peak area of interest in Figure 5a, it can be seen that the CNN is able to effectively reconstruct the peak. However, SG39 smooths the peak formation and thus reduces the intensity, while SG57 does not appear to change the raw spectrum in the peak region. From the peak area of interest for the less noisy spectrum shown in Figure 5b, it can be observed that both the CNN and SG57 effectively preserves the peak height. In the smooth region that has been enlarged on the right hand side of the image, for both cases it is shown that the CNN is able to effectively smooth the spectrum to a level that is comparable to SG39 and superior to SG57. This demonstrates that by using the CNN denoising algorithm, it is possible to achieve the benefits of both SG filters simultaneously and, in some cases, to exceed their performance.

**Figure 6:**
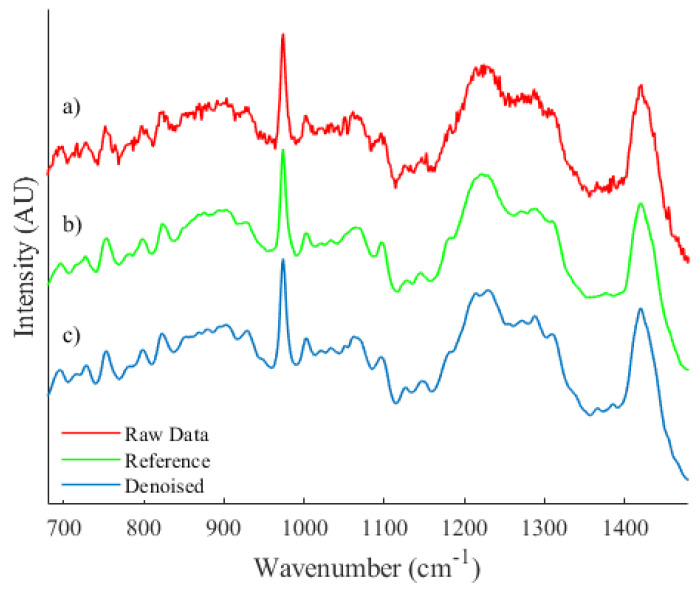
A qualitative comparison between (**a**) an unprocessed spectrum recorded from a biological sample, (**b**) a reference spectrum and (**c**) the resulting denoised spectrum.

As an example of the algorithms applicability to spectra from disparate sources, a qualitative comparison of a spectrum recorded from a biological source was performed and is shown in Figure 6. A spectrum was recorded from a prostate cancer cell line (PC3) fixed to a calcium fluoride substrate with 785 nm laser illumination and additional information on the sample preparation and system parameters may be found at Ref. [8].

Let it be noted that spectrum (b), as shown in Figure 6, is an estimation of expected collected irradiance that has been achieved by taking the average across a dataset of spectra. Due to the inherent heterogeneity of cell spectra, minor differences in the spectral profile that are unique to individual spectra within the dataset will be present and therefore a quantitative comparison could not be performed. However, the proposed algorithm is capable of quite accurately smoothing areas of high noise while preserving and effectively reconstructing local features.

These results also demonstrate that the network is capable of denoising experimental spectra from multiple sources. It is capable of recognising peak formations and prioritizing the preservation of these features while still reducing visible noise. From these results, it may be concluded that the CNN algorithm would be most appropriate for low-light or high throughput applications that would inherently collect spectra with a low SNR, i.e., SNR < 50. It may also be used to increase the throughput of applications whereby a reduced acquisition time could be compensated for by utilizing the CNN algorithm in the post-processing procedure.

## 7. Conclusions

This paper demonstrates that it is possible to train a convolutional neural network using artificial datasets of diverse spectral profiles in order to develop a denoising algorithm that may be applied to datasets comprised of spectral profiles that the network has not been specifically trained to recognize.

By creating a custom loss function through a weighted combination of global and local Mean Square Error, the performance of the algorithm increased significantly in terms of the SNR product, demonstrating the capability of the algorithm to effectively balance between smoothing the spectrum and preserving the peaks.

Spectra collected under experimental conditions that inherently produce low SNR spectra could be improved by as much as four times compared to their raw versions based on the improvements indicated by the quadrupling of the SNR product (effective doubling of SNR both globally and locally) that were evaluated on experimental data in this paper. Due to the square root relationship of the standard deviation of shot noise with respect to the collected irradiance (as it is modeled by a Poisson distribution), this improvement in SNR is analogous to recording spectra for longer exposures. Therefore, the low-SNR spectra that have been processed using the proposed algorithm will have an SNR equivalent to that of a spectrum collected using four times the acquisition time. For this reason, utilizing this algorithm in the post processing procedure for low light applications or for applications where high throughput is a priority would be advantageous. 

## Figures and Tables

**Table 1:** Network structure.

Convolutional Unit No.	No. of Filters	Filter Width	# Parameters	Output Size
1	256	9	2560	600×256
2	128	5	163,968	600×128
3	64	5	41,024	600×64
4	1	9	577	600×1
5	1	600	601	600×1

## Data Availability

The algorithm presented in this paper along with a method for generating simualted training data may be accessed through github at the following link: https://github.com/bryanhennelly/CNN-Denoiser-SENSORS (accessed on 5 July 2021).
